# Integrating Metabolic, Perfusion, and Microstructural Parameters for Quantitative Neuroimaging in Rare Neurodegenerative Diseases: A Hybrid PET/MRI Approach

**DOI:** 10.3390/diagnostics16132104

**Published:** 2026-07-05

**Authors:** Joachim Strobel, Hans-Peter Müller, Laura Michelberger, Anastasia Nosanova, Wolfgang Thaiss, Karl Georg Haeusler, Jochen H. Weishaupt, Kornelia Kreiser, Ambros J. Beer, Meinrad Beer, Jan Kassubek, Nico Sollmann

**Affiliations:** 1 Department of Diagnostic and Interventional Radiology, University Hospital Ulm, Albert-Einstein-Allee 23, 89081 Ulm, Germanymeinrad.beer@uniklinik-ulm.de (M.B.); 2 Department of Nuclear Medicine, University Hospital Ulm, Albert-Einstein-Allee 23, 89081 Ulm, Germany; 3 Department of Neurology, University Hospital Ulm, Oberer Eselsberg 45, 89081 Ulm, Germanyjan.kassubek@uni-ulm.de (J.K.); 4 Department of Diagnostic and Interventional Neuroradiology, School of Medicine and Health, TUM Klinikum Rechts der Isar, Technical University of Munich, Ismaninger Str. 22, 81675 Munich, Germany; 5 TUM-Neuroimaging Center, TUM Klinikum Rechts der Isar, Technical University of Munich, Ismaninger Str. 22, 81675 Munich, Germany

**Keywords:** positron emission tomography, magnetic resonance imaging, arterial spin labeling, diffusion tensor imaging, primary lateral sclerosis, corticobasal degeneration

## Abstract

**Background/Objectives:** The use of quantitative neuroimaging to establish objective biomarkers in neurodegenerative diseases (NDD) has attracted increasing interest over the last decade. Advanced magnetic resonance imaging (MRI) such as arterial spin labeling (ASL) and diffusion tensor imaging (DTI), as well as [^18^F]fluorodeoxyglucose ([^18^F]FDG) positron emission tomography (PET), could provide clinically meaningful biomarkers and may support differential diagnosis. The aim of this investigator-initiated, single-center, retrospective comparative study was to implement a framework for multimodal neuroimaging to evaluate cases with rare NDD, using a methodological approach that integrates metabolic, perfusion, and microstructural parameters from simultaneous FDG-PET/MRI, and to investigate its potential to facilitate diagnosis. **Methods**: Three patients with pathological motor signs (1f/2m; 63, 73, and 52 years) and 19 control subjects with subjective cognitive deficits (SCDs) underwent combined FDG-PET/MRI with pseudo-continuous ASL and DTI. Standardized uptake values (SUVs), relative cerebral blood flow (rCBF), and fractional anisotropy (FA) were calculated to identify pattern alterations in individual patients based on parameterization mapping. The final diagnosis was corticobasal degeneration (CBD, *n* = 1) or primary lateral sclerosis (PLS, *n* = 2). **Results**: At the individual patient level, disease-specific changes in defined brain regions could be demonstrated and quantified compared to control subjects. All three patients showed significantly decreased FA, primarily along parts of the course of the corticospinal tract (CST). In the patient with CBD, asymmetric SUVR and rCBF decreases were observed, mostly overlapping with motor regions. In the two patients with PLS, SUVR revealed mostly unspecific findings (hypothetically due to a slow progression rate or due to potentially early disease stages), while ASL indicated decreased rCBF primarily overlapping within the motor cortex. Changes at the gray matter level were primarily located adjacent to changes in white matter, as indicated by the multimodal analysis approach using simultaneously acquired FDG-PET/MRI data. **Conclusions**: According to this proof-of-concept study, multimodal neuroimaging by the combination of quantitative MRI and FDG-PET has the potential to guide differential diagnosis in rare NDDs, especially if clinical diagnosis is not straightforward to achieve. Since particularly early diagnosis remains essential for patient counseling, effective treatment, and clinical management, the present framework appears helpful to be developed further until it aligns and integrates with clinical routine.

## 1. Introduction

Globally, neurodegenerative diseases (NDDs) lead to considerable restrictions in quality of life and pose a significant socio-economic burden on the healthcare system [1,2]. Timely initiation of supportive and especially disease-modifying treatments—the latter becoming increasingly available for selected diseases such as Alzheimer’s disease (AD) with monoclonal antibodies or amyotrophic lateral sclerosis (ALS) with antisense oligonucleotides (ASOs) [3,4]—requires accurate diagnosis and precise differentiation between single NDD subtypes.

Operational clinical criteria have been defined for all NDDs, but clinical diagnosis alone can be challenging and may not be sufficient for reliable differentiation, especially in early stages with only mild symptoms, in symptom overlaps between NDD subtypes, or in rare NDD entities. Even for AD, as one of the most frequent NDDs, the sensitivity and specificity of common clinical diagnostic methods in correlation with neuropathological data from post-mortem analyses ranged only between approximately 71 and 87% and 44 and 71%, respectively [5]. For less common NDDs, such as rare neurodegenerative parkinsonian syndromes like corticobasal degeneration (CBD) [6], diagnostic yield might be even more restricted with sensitivity or accuracy values below 50% for CBD, which might hold true especially at early presentation after symptom onset [7,8]. Hence, for rare NDDs, correct diagnosis can become substantially delayed, with median times from index symptoms to the differential diagnosis of atypical parkinsonian syndromes of approximately 3 to 4 years or diagnostic delays ranging from 9.1 to 27 months in ALS [9,10].

Neuroimaging can support differential diagnosis in NDDs and rule out other diseases [11,12,13]. Standard structural magnetic resonance imaging (MRI) is commonly applied; however, routine visual image reading inherently showed considerable inter-observer variability [12]. By the use of advanced MRI, including methods such as diffusion or perfusion imaging, specific spatial patterns of affected brain areas and characteristic neural signatures of diseases could be detected and quantified [12,14]. When combined with metabolic imaging from positron emission tomography (PET), multi-parametric approaches become possible, thus combining information from (micro)structure, perfusion, and metabolism. However, such integrative approaches combining advanced MRI with PET are only sparsely used in NDDs to date [13,15]. In part, this relates to the complexity of integrating neuroimaging data from different sequences and applying unified postprocessing algorithms to derive meaningful visualization and parameter quantification.

Against this background, our comparative study describes a multi-parametric neuroimaging approach for combined PET/MRI in selected patients with rare NDDs at the stage of early diagnosis, combining complementary information of brain metabolism, microstructure, and perfusion. Our aim was to provide a proof of concept for an integrated imaging-based framework that could aid initial differential diagnosis in patients with rare NDDs in early clinical stages when clinical diagnosis can be inconclusive.

## 2. Materials and Methods

### 2.1. Study Population

This monocentric study was approved by the Ethics Committee of the University of Ulm (approval code: Nr. 164/24; approval date: 26 July 2024), certifying that the comparative study complies with the Declaration of Helsinki and its amendments or equivalent ethical standards. The requirement for written informed consent was waived due to the retrospective study design.

Three patients with clinical syndromes, which could not be classified on clinical grounds (NDD001, NDD002, and NDD003), and 19 individuals with subjective cognitive deficits (SCDs), who served as an analogue of a control group, were selected from the local database, with the requirement that all participants had been investigated with the identical PET/MRI acquisition protocol on the same PET/MRI system due to clinical indications. In all cases, the initial diagnosis was equivocal; thus, PET/MRI was requested to support differential diagnosis and final diagnosis determination. In the end, the final diagnosis in the three patients was made under consideration of all available examinations and in agreement with current guidelines (e.g., consensus diagnostic criteria for primary lateral sclerosis [PLS] or CBD [6,16]).

For the group of individuals with SCDs, any NDD diagnosis was ruled out based on all available clinical and imaging data (tau biomarkers were not available in these individuals, given the low suspicion of an NDD based on all available data). The Mini-Mental State Examination (MMSE) results were in a range of 27 to 30 points for individuals with SCD. The number of included individuals with SCDs resembles the available cases identified via search of our local database regarding subjects without a diagnosis of an NDD or concurrent diagnosis regarding subjective neurocognitive impairments. None of these subjects had any known drugs targeted at neurological or psychiatric conditions.

### 2.2. Image Acquisition

The multi-parametric imaging framework consisted of PET and MRI sequences that were acquired during a ~45 min scan of dynamic PET in list mode on an integrated 3-Tesla PET/MRI scanner (with a 12-channel head coil; Biograph mMR, Siemens Healthineers, Erlangen, Germany) using dedicated postprocessing [17]. All subjects underwent the identical MRI acquisition protocol with PET acquisitions, including diffusion tensor imaging (DTI) and pseudo-continuous arterial spin labeling (pCASL) [17]. In addition, a high-resolution non-contrast-enhanced three-dimensional (3D) T1-weighted magnetization-prepared rapid gradient echo (MPRage) sequence was acquired in the same session.

### 2.3. Routine Image Processing and Visual Image Reading

All images were visually evaluated by at least two board-certified radiologists and one board-certified nuclear medicine specialist. In addition, the 3D T1-weighted data (repetition time [TR] = 1900 ms, echo time [TE] = 3.25 ms, inversion time [TI] = 900 ms, pixel spacing = 0.5 × 0.5 mm, slice thickness = 0.9 mm, number of slices = 192) were processed with mdbrain software (version 4.13.0; mediaire GmbH, Berlin, Germany) for global and regional brain volumetric assessments, providing deviations from normal volumetry in comparison to a heterogeneous database considering age, sex, and intracranial volume (approximately 8000 datasets).

### 2.4. Image Processing of the Multi-Parametric Framework

The pre- and post-processing steps of the analyses were performed using the Tensor Imaging and Fiber Tracking (TIFT) software (version 2025) [17,18]. Non-linear spatial normalization was performed using study-specific templates (oriented to the Montreal Neurological Institute [MNI] stereotaxic standard space and using the “Colin brain template” [19,20]), preserving directional information in case of DTI data processing [18]. This normalization procedure has been established for DTI in previous work, and it has been adapted to the normalization of pCASL data and PET data, each with modality- and study-specific templates [17].

#### 2.4.1. PET Data

Details of PET acquisition have already been reported in a previous study [17]. Patients were placed in a darkened, soundproof room approximately one hour before the examination. Weight-adapted doses were used (3.0 MBq/kg; range of approximately 200 MBq to 500 MBq, with a half-life of approximately 110 min), and the radiopharmaceutical was applied after ~30 min, thus allowing for additional ~30 min of uptake before the examination was conducted. List-mode PET data were acquired (236 ± 50 MBq) and were reconstructed from acquisition minute 35 to minute 45 to one image volume (344 × 344 × 127 matrix) using Siemens SyngoVia (VB60S; Siemens Healthineers, Erlangen, Germany). The reconstructed PET images had a spatial resolution of ~4.2 × 4.2 × 2.0 mm^3^ (matrix 172 × 172 × 127). Standardized uptake values (SUVs) were initially derived from the PET data by normalization to injected dose and patient body weight. Subsequently, SUV ratio (SUVR) maps were computed by further normalizing regional SUVs to the mean whole-brain uptake, which served as the reference. All subsequent analyses were based on the obtained SUVR values. The resulting maps (interpolated onto a 1 mm isotropic voxel grid) were used for spatial smoothing with an isotropic 8 mm full width at half maximum (FWHM) Gaussian kernel to obtain a balance between sensitivity and specificity, and to accommodate individual anatomical variability.

#### 2.4.2. PCASL Data

As used in a previous study, the sequence had the following parameters [17]: TR = 4110 ms, TE = 37.9 ms, permuted post-labeling delays (PLDs) of 1.5 s / 1.8 s / 2.0 s, acquisition of 36 label and control pairs (i.e., 12 label/control pairs per each PLD), parallel imaging acceleration factor of 2 along the phase-encoding direction with generalized autocalibrating partial parallel acquisition (GRAPPA) reconstruction, refocusing flip angle of 120 degrees, bandwidth of 2170 Hz/pixel, and spatial resolution of ~3.9 × 3.9 × 3.6 mm^3^ (matrix of 64 × 64 × 49). After motion correction, maps of CBF were generated from the pCASL data; each voxel’s CBF values were normalized to the average CBF across a whole-brain mask to standardize intensity [21]. Data were then interpolated onto a 1 mm isotropic voxel grid to enhance spatial resolution for subsequent analyses, and relative CBF (rCBF) maps were computed accordingly. Given that images were obtained at three PLDs, rCBF maps from the single PLDs were averaged. The normalized rCBF maps were smoothed using an isotropic 8 mm FWHM Gaussian filter to match the intrinsic smoothness of the data [22].

#### 2.4.3. DTI Data

Whole-brain DTI was performed axially, using a two-dimensional (2D) single-shot echo planar imaging (EPI) sequence with 64 gradient directions (b = 1000 s/mm^2^ and b = 0 s/mm^2^) and the following parameters, as outlined previously [17]: TR = 13,500 ms, TE = 94 ms, resolution of ~2.1 × 2.1 × 1.8 mm^3^ (matrix of 108 × 108 × 86), parallel imaging with an acceleration factor of 2 along the phase-encoding direction (GRAPPA). The bandwidth was set at 1446 Hz/pixel. An established quality control protocol was followed to compensate for corrupted gradient directions as well as motion artifacts prior to correction of eddy current-induced geometric distortions [23]. Data were interpolated onto a 1 mm isotropic voxel grid, fractional anisotropy (FA) maps were calculated from MNI-normalized DTI data, and a Gaussian smoothing filter of 8 mm FWHM was applied to the normalized individual FA maps to balance sensitivity and specificity. The FA maps were corrected for age according to a standardized protocol [24].

#### 2.4.4. Whole Brain-Based Spatial Statistics and Region of Interest-Based Analyses

To estimate whether the patients (NDD001, NDD002, and NDD003) of this study showed alterations in the different parameters (i.e., SUVR from PET, FA from DTI, rCBF from ASL), a voxel-wise statistical comparison to the SCD group was performed using whole brain-based spatial statistics (WBSS). Statistical results by the method of Crawford and Howell were corrected for multiple comparisons using a false-discovery rate (FDR) at a significance level of *p* < 0.05 [25,26]. Further reduction in the alpha error was performed by a spatial correlation algorithm that eliminated isolated voxels or small isolated groups of voxels in the size range of the smoothing kernel, leading to a threshold cluster size of 256 voxels (256 mm^3^) [17].

#### 2.4.5. Definition of Regions of Interest

The selection of regions of interest (ROIs) was based on established areas of hypometabolism, hypoperfusion, and/or microstructural changes identified in the literature and relevant to CBD or ALS/PLS. Specifically, for CBD, PET showed hypometabolism in gray matter (GM) of temporal areas [27,28], and DTI showed FA reductions especially in pre- and postcentral gyri [29]. At the group level, hypometabolism in ALS was reported in the central/postcentral gyri [30,31], microstructural degeneration in ALS was reported especially along the corticospinal tract (CST) [32,33], and perfusion abnormalities in ALS patients showed a mixed picture of CBF decreases in a series of brain regions including frontal regions, the prefrontal cortex, motor cortex, and post-central gyri, as well as temporal and fronto-temporal regions [34]. The ROIs were located at locations of coincidence of previous meta-analyses (e.g., DTI [35] and PET [36], no suitable meta-analysis for ASL available). The ROI analyses complemented the neuroimaging patterns of the voxel-wise analyses and could help to distinguish whether the imaging patterns were individual (i.e., occurring in more than one subject) or disease-specific findings.

The ROI analyses were performed by arithmetically averaging the relevant parameter (i.e., SUVR from PET, FA from DTI, rCBF from ASL) for each modality and each subject within a given spherical region, yielding mean ROI-related average values (i.e., <SUVR>_ROI_, <FA>_ROI_, <rCBF>_ROI_). The radius of the spherical ROIs was set to achieve a good balance between sensitivity and specificity. For DTI analysis, only voxels with FA values greater than 0.2 were considered. For SUVR and rCBF, no thresholds were applied.

In the following, significance is used for voxels that (after the Crawford–Howell test) survived FDR adjustments at a level of 0.05 and subsequently formed clusters that had a minimum extension of 256 voxels.

## 3. Results

### 3.1. Participant Characteristics

Three patients with initially unclassified NDD (NDD001: 63 years, male; age NDD002: 73 years, female; NDD003: 52 years, male) and 19 subjects with SCD (9f/10m; mean age 55.0 ± 12.8 years, age range 26.3–74.1 years) were included and analyzed (Table 1). Those 19 subjects with SCDs served as an analogue of a control group for this study. The final diagnosis in the three patients was PLS (NDD001), CBD (NDD002), and PLS (NDD003), with the distinct diagnoses being initially equivocal and referral for PET/MRI by specialized neurologists for diagnostic support.

In NDD001, a slowly progressive gait disorder over approximately 2.5 years prior to the PET/MRI acquisition was initially observed with symmetrical involvement, together with fine motor skill impairments in the right hand. Clinically, a spastic gait could be observed with brisk jerks and slight symmetrical rigidity in the legs, with no direct signs of lower motor neuron involvement. In NDD002, symptoms started approximately 4 years ago, with unilateral numbness in the left hand, followed by dystonia, apraxia, and left-sided muscle tone increase with combined rigidity and spasticity. In NDD003, slowly progressive motor strength impairment for about 24 months prior to the PET/MRI acquisition of the upper extremities has been observed, paired with fine motor skill impairments bilaterally and gait insecurity with slight combined spastic and rigid muscle tone in the arms (pronounced) and legs. The respective initial diagnosis from the three patients’ clinical presentation was not entirely clear, including differential diagnoses such as motor neuron disease, myelopathy, or basal ganglia/parkinsonian disorders.

**Figure 1:**
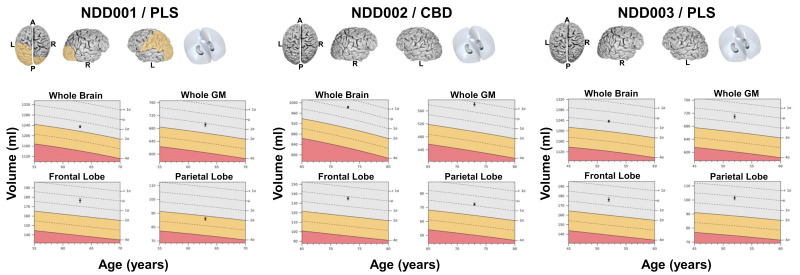
The anatomical three-dimensional (3D) T1-weighted images in three patients with neurodegenerative diseases (NDDs; primary lateral sclerosis [PLS] for NDD001, corticobasal degeneration [CBD] for NDD002, and PLS for NDD003) were processed for global and regional brain volumetric assessment, indicating deviations from normal volumetry versus a heterogeneous database (in ml, considering age, sex, and intracranial volume). Results for volumetric assessment are shown for the whole brain, whole gray matter (GM), frontal lobe, and parietal lobe. Orange color indicates reduced volumes (−2σ), and red color indicates severely reduced volumes (−4σ). A—Anterior, P—Posterior, L—Left, R—Right for the brain templates, GM—Gray matter.

Visual image reading of MRI did not reveal any gross structural abnormalities or clearly advanced brain atrophy. Automated brain volumetry also did not detect any global or regional deviation of volumetric measures from normal volumetry in two patients (NDD002 and NDD003), while slightly decreased volumes were found in one patient (NDD001) for the left-hemispheric parietal lobe and the occipital lobes (Figure 1).

### 3.2. Whole Brain-Based Spatial Statistics

**Figure 2:**
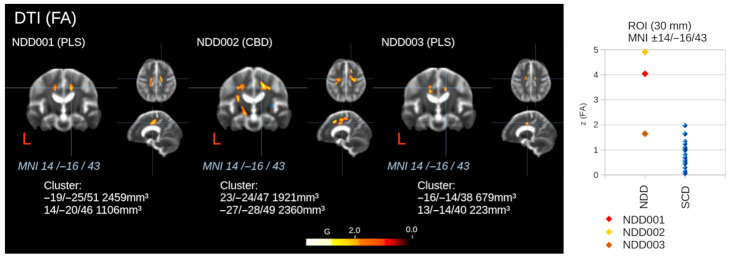
Voxel-wise statistical comparisons in three patients with neurodegenerative diseases (NDDs; primary lateral sclerosis [PLS] for NDD001, corticobasal degeneration [CBD] for NDD002, and PLS for NDD003) regarding fractional anisotropy (FA) from diffusion tensor imaging (DTI) by whole brain-based spatial statistics (WBSS) against 19 individuals with subjective cognitive deficits (SCDs), considering false-discovery rate (FDR) adjustments. Maximum cluster coordinates (in Montreal Neurological Institute [MNI] space) and sizes are provided. Hot color clusters indicate decreases, while a cold color cluster (in NDD002) indicates increases in FA. Additionally, region of interest (ROI) analysis results for a ROI in the upper corticospinal tract (CST) are shown in comparison to the subjects with SCDs. “L” indicates the left hemisphere.

**Figure 3:**
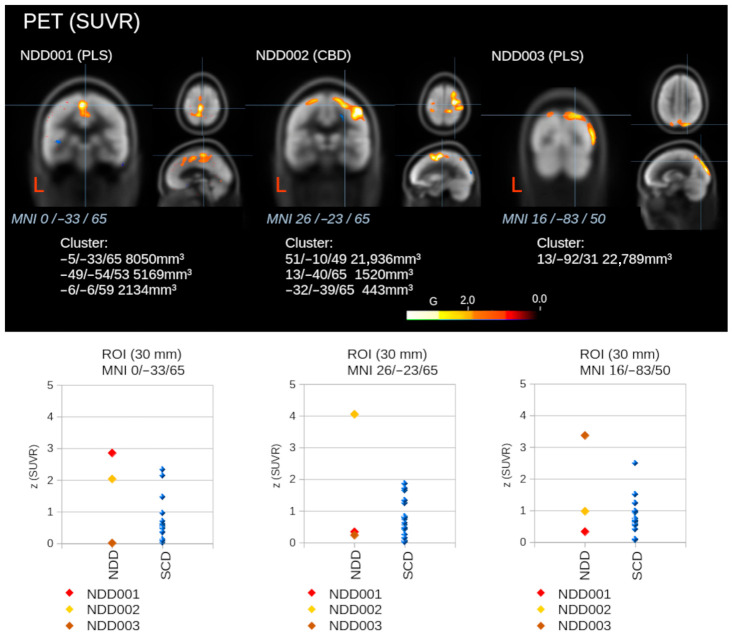
Voxel-wise statistical comparisons in three patients with neurodegenerative diseases (NDDs; primary lateral sclerosis [PLS] for NDD001, corticobasal degeneration [CBD] for NDD002, and PLS for NDD003) regarding standardized uptake value ratio (SUVR) from positron emission tomography (PET) by whole brain-based spatial statistics (WBSS) against 19 individuals with subjective cognitive deficits (SCDs), considering false-discovery rate (FDR) adjustments. Maximum of cluster coordinates (in Montreal Neurological Institute [MNI] space) and sizes are provided. Hot color clusters indicate decreases, while cold color clusters (in NDD001 and NDD002) indicate increases in SUVR. Additionally, region of interest (ROI) analysis results for ROIs at locations of maximum alteration clusters are shown in comparison to subjects with SCDs, respectively. “L” indicates the left hemisphere.

**Figure 4:**
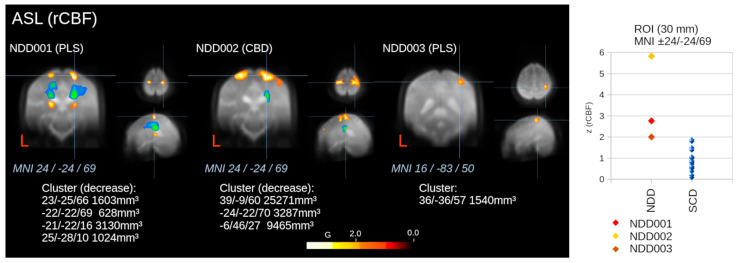
Voxel-wise statistical comparisons in three patients with neurodegenerative diseases (NDDs; primary lateral sclerosis [PLS] for NDD001, corticobasal degeneration [CBD] for NDD002, and PLS for NDD003) regarding relative cerebral blood flow (rCBF) from arterial spin labeling (ASL) by whole brain-based spatial statistics (WBSS) against 19 individuals with subjective cognitive deficits (SCD), considering false-discovery rate (FDR) adjustments. Maximum of cluster coordinates (in Montreal Neurological Institute [MNI] space) and sizes are provided. Hot color clusters indicate decreases, while cold color clusters (in NDD001 and NDD002) indicate increases in rCBF. Additionally, region of interest (ROI) analysis results for ROIs in the motor cortex are shown in comparison to subjects with SCD. “L” indicates the left hemisphere.

Individual voxel-wise statistical comparisons in the three patients (NDD001, NDD002, and NDD003) versus the subjects with SCDs for SUVR, rCBF, and FA by WBSS are displayed as heat maps (Figure 2, Figure 3 and Figure 4).

Regarding WBSS of FA maps from DTI, all NDD patients showed an FA decrease (i.e., microstructural impairment), especially in the upper CST, which was most pronounced in NDD002 (Figure 2). The scatter plot in Figure 2 shows the absolute z-values in ROIs in the upper CST. NDD001 and NDD002 showed an absolute z-value (i.e., FA decrease) distinct from SCDs, whereas NDD003 showed an overlap with two SCDs, nevertheless significantly different from the average in the SCD group. This effect could be a result of using a group of subjects with SCDs instead of purely healthy controls. In detail, FA values were decreased along parts of the CST of both hemispheres in NDD001 and NDD003, yet with larger clusters of FA decreases and higher corresponding z(FA) increases in NDD001. In NDD002, also relatively large clusters of FA decreases were found primarily along the CST of both hemispheres, yet with an asymmetric pattern.

In WBSS of SUVR, NDD001 showed a cluster with significant hypometabolism close to the midline and in the central and postcentral gyrus, while NDD002 showed a cluster with significant hypometabolism in frontal and also central areas (bihemispheric, more pronounced within the right hemisphere), and NDD003 showed a cluster with significant hypometabolism mostly in the superior parietal lobes (bihemispheric, more pronounced within the right hemisphere) (Figure 3). The scatter plots in Figure 3 show the absolute z-values in ROIs at different positions (guided by a significant cluster in the respective NDD patient), thereby demonstrating the values of the other two patients in a ROI that was significant in the respective patient. Basically, these significant z-deviations were present only for the respective single NDD patient; the further two NDD patients showed no differences in z-values for these ROIs. Regarding related z(SUVR) values, a mixed pattern was observed for the three patients, yet with individual cluster-specific increases for single ROIs when compared to the individuals with SCD. Specifically, corresponding ROI analysis for a ROI in the central or postcentral gyrus showed increases in z(SUVR) for NDD001 or NDD002 (Figure 3).

In WBSS of rCBF, NDD001 and NDD002 showed significantly reduced perfusion primarily at or close to the motor cortex (bihemispheric), whereas NDD003 showed only a small cluster of reduction at the motor cortex (Figure 4). The scatter plot in Figure 4 shows the absolute z-values in ROIs in the motor cortex, with NDD001, NDD002, and NDD003 being distinct from individuals with SCDs (significant rCBF reductions). More specifically, in NDD001, mostly symmetric bihemispheric clusters of rCBF decreases were found along the central gyrus, yet showing more laterally expanding alterations when compared with PET-derived SUVR (Figure 4). In NDD002, relatively large bihemispheric but asymmetric clusters of rCBF decreases were found, primarily along the central gyrus, with more pronounced clusters within the right hemisphere (Figure 4). In contrast, only a small cluster of rCBF decrease was found within the right hemisphere in NDD003, overlapping with the right-hemispheric central gyrus (Figure 4). Especially in NDD001 and to a lesser extent also in NDD002, clusters with increases in rCBF were found (in both hemispheres for NDD001 and in the right hemisphere for NDD002). The corresponding ROI analyses at the motor cortex showed significant rCBF reductions with corresponding z(rCBF) increases in all patients (Figure 4).

## 4. Discussion

Our integrated multimodal neuroimaging framework might allow us to systematically investigate the neural signatures of metabolic, perfusion, and microstructural changes in patients with rare NDDs. Multimodal neuroimaging showed characteristic regional decrease patterns (predominantly in the GM) in PET-derived glucose metabolism (i.e., SUVR) and ASL-derived rCBF, and alteration patterns (preferably in the white matter [WM]) in DTI-derived FA. At the individual level, an initial estimation of subject-specific changes in defined brain regions (hypothetically disease-related) could thus be demonstrated and quantified. However, given the small sample size, rather than showing disease-specific imaging patterns or diagnostic utility in representative cohorts, this study suggests feasibility and allows generation of hypotheses rather than demonstrating final clinical applicability. In contrast to a previous related study at the group level [17], this study may pave the way from studies at the group level to the possibility of individual imaging-based categorization/analysis by comparing single-subject data to a group of subjects/patients.

A timely and accurate differential diagnosis between NDD subtypes is highly important in the light of the constantly evolving therapeutic portfolio, and this may be facilitated by neuroimaging techniques [11,12,13]. Especially quantitative and multi-parametric approaches may be suited to capture the multifaceted alterations of NDD subtypes, as represented by the herein used framework integrating metabolic, perfusion, and microstructural parameters from simultaneous FDG-PET/MRI acquisitions. Notably, the patients investigated showed a clinical presentation that was not clearly attributable to a specific disease entity in the first place, and they were finally diagnosed with an atypical parkinsonian syndrome (CBD, NDD002) or an atypical motor neuron disease (PLS, NDD001 and NDD003), respectively. In this regard, for CBD, hypometabolism in perirolandic regions and the thalamus and markedly in the basal ganglia can be observed by FDG-PET according to previous work, with a characteristic dominance towards the clinically more affected hemisphere [27,37]. For the ALS variant PLS, typically primary motor cortex hypometabolism can be observed on FDG-PET, together with hypometabolism in projection on the course of the CST [38,39,40]. For perfusion imaging, ASL has been used only rarely, with previous work indicating CBF asymmetry in the perirolandic area and parietal cortex in patients with corticobasal syndrome [41]. Regarding motor neuron diseases, hypoperfusion was found especially for motor regions, but also for non-motor regions such as fronto-temporal regions [34]. For WM microstructure as evaluated by DTI, patients with corticobasal syndrome showed FA decreases in the sensorimotor projections of the cortical hand areas, among other regions [42,43]. In ALS and also in PLS, DTI has been used to predominantly investigate the CST, with FA being typically reduced at baseline or progressively over time [44,45]. Yet, previous work mostly used one imaging modality in isolation, thus being unable to reveal disease-related alterations in multiple domains at the same time [27,38,40,41,42,43,44,45]. Our study, however, applied combined FDG-PET, ASL, and DTI to rare and atypical variants of NDDs, thereby paving the way for the identification of hypothetical disease-related changes. In a general context, the definition of ROIs—which in this study was based on established areas of hypometabolism, hypoperfusion, and/or microstructural changes identified in the literature and conceptualized by WBSS analyses—cannot yet be considered final and should be refined in studies involving large patient cohorts; further definitions of pathologically indicated ROI locations can be found in previous related work [17].

For NDD001, in whom diagnosis remained initially vague with spastic and rigid muscle tone versus PLS in the end, our multi-parametric framework revealed decreased SUVR in one cluster close to the midline in the central and postcentral gyrus, while rCBF maps were in agreement with the location of the central gyrus bilaterally, but showing more laterally expanding alterations overlapping with the “hand knob” as the anatomical location of primary motor function. Downstream effects of those impairments seem to be reflected by bilateral FA decreases along parts of the course of the CST. In this case, all three methods from simultaneous acquisition point to a marked disease involvement of the motor system, both on the GM and WM level. Consideration of ASL and DTI in addition to FDG-PET may enhance diagnostic confidence with high complementary yield. Notably, changes within the motor system were most markedly highlighted by ASL decreases, and the rather symmetric alterations favored a PLS diagnosis over CBD from a neuroimaging point of view. Yet, also relatively large clusters of bihemispheric rCBF increases were found, primarily located within subcortical areas and WM. While large-scale studies on ASL specifically in patients with PLS are mostly lacking, those changes may indicate complex patterns in the disease course and pathophysiology of PLS as expressed by rCBF increases, which may potentially represent compensatory mechanisms at the subcortical and WM level that are related to the marked rCBF decreases predominantly at the cortical level in NDD001.

In NDD002 with a final diagnosis of CBD, SUVR was decreased at the right central gyrus, partially also overlapping with the rCBF maps, which showed a large right-hemispheric and smaller left-hemispheric cluster at the central gyrus. Furthermore, FA maps showed bihemispheric decreases along parts of the CST. In this regard, the findings from ASL and DTI seem remarkable in that they highlight bihemispheric yet asymmetric changes in the motor system, with bihemispheric changes being relatively widespread. While CBD is characteristically asymmetric (especially early in the disease course), it is bihemispheric in the underlying pathology, involving both sides of the brain along the progression trajectory [46,47]. Even in the case of unilateral symptoms, the underlying tau protein accumulation is commonly widespread across both hemispheres and the basal ganglia, also affecting WM structures [48,49,50].

In NDD003 with a final diagnosis of PLS, small parietal clusters of decreased SUVR were observed, while rCBF maps showed one cluster with decreases in the right central gyrus, and FA maps indicated discrete bihemispheric decreases especially in the upper/central CST. In this case, rather subtle changes were revealed, which might go unnoticed or classified as unspecific when solely using information from one imaging modality or visual image readings. The findings were more subtle than in NDD001, hypothetically reflecting the earlier disease course. Notably, findings can also be negative for FDG-PET in PLS, which typically has a slower disease course as compared to ALS [51]. Overall, while from PET/MRI acquisitions the structural standard sequences are oftentimes used for mere anatomical co-registration, the adjunct of advanced sequences such as ASL and DTI may facilitate correct diagnoses also in cases where SUVR alterations are minor or not entirely specific, and they could also improve diagnostic confidence by delivering complementary information.

Given that perfusion imaging by ASL is a modality with a limited number of applications to ALS/PLS as well as CBD so far, the results of the current comparative study add to the previous literature, which has revealed abnormalities in perfusion (especially including hypoperfusion) in primary motor areas [34,41]. These findings could be confirmed in our study, and it could be demonstrated that the PET- and ASL-derived significant clusters slightly differed from each other, although they were located adjacent to each other or partially overlapped (mostly along the motor cortices). Yet, distinct individual spatial patterns and also bihemispheric alterations have been revealed in our patients, which may further highlight the complementary potential. In addition, DTI demonstrated significant reductions in FA in WM regions predominantly adjacent to the hypometabolic and hypoperfused GM areas, so that WM alterations could be considered to be closely tied to GM impairment. Those results were obtained in comparison to individuals with SCDs. Statistically, the group of individuals with SCDs represents a group of subjects with no conspicuousness on PET/MRI and a relatively normal distribution of PET/MRI-derived analysis parameters; even if one subject would be in a very early stage of a potential NDD, the contribution to the group-level parameter would be in the order of ~6% and, thus, should not significantly influence the statistical comparison of the respective patient with NDD versus the SCD group.

The present ASL analyses processed each PLD separately and subsequently averaged the resulting CBF maps, that way ensuring stability within the context of this study; however, this does not fully utilize the potential of multi-delay acquisitions. Multi-PLD perfusion imaging would make it possible to determine the most efficient multi-PLD acquisition protocol in terms of the number of label/control pairs and could further refine ASL results. Model-based approaches that jointly estimate CBF and arterial transit times (ATTs) have been shown to provide more physiologically accurate perfusion estimates, particularly in samples with prolonged or heterogeneous ATTs, such as older patients or patients with cerebrovascular diseases [21,52]. Thus, multi-PLD perfusion imaging that includes rather short PLDs (<1.0 s) and rather long PLDs (>2.5 s) is necessary to provide sufficient information to estimate ATT for calculating adjusted CBF maps [53].

Regarding limitations, our study included only a very limited number of exemplary cases, diagnosed with rare and atypical NDD entities. Of note, the main intention of this methodological investigation was to propose a modular framework for parameterized multimodal neuroimaging, which could facilitate differential diagnosis especially in clinically complex cases and rare NDDs. By use of this methodological approach, a detailed association assessment of neuroimaging patterns and clinical phenotypes could be performed in the future. Although observations from the present study are not representative, the findings suggest that a combined PET/MRI framework could help to improve phenotype characterization in NDDs at an individual level. Since this study was a retrospective investigation, a group of individuals with SCDs was used to determine the location of the ROIs and for the comparisons, rather than a group of completely healthy control subjects. This SCD group may have included individuals with preclinical neurodegenerative changes or other neurological/psychiatric comorbidities (though without detectable pathological findings on PET). However, entirely healthy subjects typically do not undergo PET examinations in clinical routine, due to its invasiveness and radiotracer-related radiation exposure. From a statistical point of view, even if one subject would be in a very early stage of a neurodegenerative disease, the contribution to the group-level parameter would be in the order of ~6%. Nevertheless, the methodology of this study should ideally be validated in the future using age- and sex-matched healthy controls. Furthermore, the volumetric assessments from T1-weighted images based on commercially available software were derived from comparisons to control datasets as normative data. However, these datasets may not necessarily include balanced geographic, ethnic, or demographic composition, and the educational status was unknown. Thus, local controls with homogeneous sequences from the same MRI scanner and elaborate clinical information may have been used for more exact reference comparisons. As a final limitation, it has to be noted that when the framework is applied to patients whose neuroanatomy diverges substantially from the template, normalization errors could systematically affect parameter quantification in ways that the current analysis cannot detect.

## 5. Conclusions

Using a comparative approach, we describe a multi-parametric neuroimaging approach for combined PET/MRI, integrating complementary information of brain metabolism, microstructure, and perfusion, and its application to selected patients with rare NDDs of the motor system at the stage of initial diagnosis. That way, this approach could potentially provide relevant neuroimaging-related information in selected cases, and further verification is needed in larger prospective cohorts. However, the methodology provides a clinically translatable model that aligns neuroimaging biomarkers with the pathophysiology and clinical findings in different rare NDDs. Hence, this framework could provide additional information from neuroimaging studies to aid in the clinical diagnosis of rare NDDs.

## Figures and Tables

**Table 1:** Characteristics for patients with rare neurodegenerative diseases (NDDs) and individuals with subjective cognitive deficits (SCDs). UPDRS—Unified Parkinson’s Disease Rating Scale, ALS-FRS-R—Revised Amyotrophic Lateral Sclerosis Functional Rating Scale (0 worst—48 best), MMSE—Mini-Mental State Examination.

	Age (in Years)	Sex	Disease Duration (in Years)	Primary Symptoms	Clinical Scores
**N** **DD001**	63	m	~2.5	Slowly progressive spastic gait disorder	ALS-FRS-R 30
**NDD002**	73	f	~4.0	Left-sided dystonia, apraxia, combined rigidity and spasticity	UPDRS III 32
**NDD003**	52	m	~2.0	Spastic tetraparesis with fine motor skill impairments bilaterally and gait insecurity	ALS-FRS-R 42
**SCD**	55 ± 12.8	9f/10m	n.a.	None	MMSE 27–30

## Data Availability

The data that support the findings of this study are in part available from the corresponding author, but restrictions may apply to the availability of these data. In addition, in the event that some of the data can be shared, an official data sharing agreement with the University Hospital Ulm has to be obtained.
